# Comparing the physiochemical parameters of three celluloses reveals new insights into substrate suitability for fungal enzyme production

**DOI:** 10.1186/s40694-017-0039-9

**Published:** 2017-11-03

**Authors:** Lara Hassan, Manfred J. Reppke, Nils Thieme, Steffen A. Schweizer, Carsten W. Mueller, J. Philipp Benz

**Affiliations:** 1 HFM, TUM School of Life Sciences Weihenstephan, Technical University of Munich, Freising, Germany; 2 Chair of Soil Science, TUM School of Life Sciences Weihenstephan, Technical University of Munich, Freising, Germany

**Keywords:** Microcrystalline cellulose, Powdered cellulose, Cellulase production, Cellulose crystallinity, *Neurospora crassa*, *Trichoderma reesei*, RUT-C30

## Abstract

**Background:**

The industrial applications of cellulases are mostly limited by the costs associated with their production. Optimized production pathways are therefore desirable. Based on their enzyme inducing capacity, celluloses are commonly used in fermentation media. However, the influence of their physiochemical characteristics on the production process is not well understood. In this study, we examined how physical, structural and chemical properties of celluloses influence cellulase and hemicellulase production in an industrially-optimized and a non-engineered filamentous fungus: *Trichoderma reesei* RUT-C30 and *Neurospora crassa*. The performance was evaluated by quantifying gene induction, protein secretion and enzymatic activities.

**Results:**

Among the three investigated substrates, the powdered cellulose was found to be the most impure, and the residual hemicellulosic content was efficiently perceived by the fungi. It was furthermore found to be the least crystalline substrate and consequently was the most readily digested cellulose in vitro. In vivo however, only RUT-C30 was able to take full advantage of these factors. When comparing carbon catabolite repressed and de-repressed strains of *T. reesei* and *N. crassa*, we found that *cre1*/*cre*-*1* is at least partially responsible for this observation, but that the different wiring of the molecular signaling networks is also relevant.

**Conclusions:**

Our findings indicate that crystallinity and hemicellulose content are major determinants of performance. Moreover, the genetic background between WT and modified strains greatly affects the ability to utilize the cellulosic substrate. By highlighting key factors to consider when choosing the optimal cellulosic product for enzyme production, this study has relevance for the optimization of a critical step in the biotechnological (hemi-) cellulase production process.

**Electronic supplementary material:**

The online version of this article (10.1186/s40694-017-0039-9) contains supplementary material, which is available to authorized users.

## Background

Due to their wide applicability, the demand for cellulases and hemicellulases is constantly increasing. Currently, these enzymes are used in the processing of food and animal feed, in the textile and laundry industries, for pulping and paper production, as well as for the biofuels industry [1]. The overall technical enzymes market is projected to reach a value of 1.27 billion USD in 2021, with the bioethanol application predicted to be the fastest-growing section [2]. The goal here is to efficiently convert sustainably produced lignocellulosic feedstocks to fermentable sugars for the production of biofuels, but also other products of the biorefinery. Due to the high recalcitrance of cellulose, this process requires high enzyme loadings, warranting research efforts aiming to increase enzyme yields and decrease the production costs.

Cellulose is composed of unbranched chains with repeating ß-1,4-linkages of only d-glucose units. Many parallel glucan chains form tight microfibrils held together by hydrogen bonds, rendering the surface of cellulose highly hydrophobic and recalcitrant to enzymatic attack [3–6]. Traditionally, the fine structure of cellulose is described in a simplistic two-phase model, in which highly ordered regions are classified as crystalline and less well-ordered regions as amorphous [7]. Moreover, cellulose in the natural setting is embedded in a matrix of hemicelluloses and lignin, adding structural support and protection [8, 9]. The major hemicelluloses in hardwoods and grasses are xylans and mixed-linkage glucans, while (galacto)glucomannans dominate in softwoods [10–12].

Cellulases are commonly produced by fermentation of lignocellulosic substrates with microorganisms, such as bacteria or filamentous fungi. Microcrystalline celluloses (MCCs) have been used as excipients in the pharmaceutical industry for decades, but are also used as cellulase-inducing substrates due to their purity, availability and ease of use. MCCs are usually prepared by treatment of cotton linters or wood pulp with dilute mineral acid to hydrolyze and extract the amorphous regions of cellulose as well as hemicelluloses, lignin and pectin [13, 14]. The result is a partially depolymerized cellulose with a limited degree of polymerization in the form of colloidal crystallites that can aggregate and agglomerate to particle sizes of usually between 20 and 200 µM [15]. MCCs are derived from various sources, such as hardwoods and softwoods. Various products from the international market have been shown to differ in their characteristics regarding crystallinity, monosaccharide composition, and particle size [16–19]. Moreover, batch-to-batch variability has been shown to have an equally strong impact on the MCC properties [20].

The filamentous ascomycete *T. reesei* (teleomorph *Hypocrea jecorina*) has become the preferred organism for the production of cellulases [21–23], one of the best known and publicly available strains being RUT-C30 of the Rutgers lineage derived from screens for hyper-cellulase production after rounds of classical mutagenesis [24, 25].

*Trichoderma reesei* has also been instrumental in the elucidation of the molecular factors underlying the perception and degradation of cellulose in filamentous fungi [26]. The general principle of induction and repression governing the response is conserved as in all microorganisms, but varies in its implementation between fungi (for a review, see [27]). In *T. reesei* (and species of the genus *Aspergillus*), the transcription factor (TF) XYR1/XlnR is the major regulator of the cellulolytic and hemicellulolytic response, even though recently ACE3 was described in *T. reesei* as a novel master regulator of cellulase expression and a modulator of xylan degrading enzyme expression [28]. In other fungi, such as in the genetic model system *N. crassa*, the XYR1 homologs only modulate production of cellulases and are mainly required for the induction of hemicellulases (for recent reviews, see [29, 30]). Instead, two other conserved TFs in tandem govern the response to cellulose: CLR-1 and CLR-2 [31, 32]. The function of CLR-2 does not seem to be strictly conserved in *T. reesei* [28], but is in other fungi such as in *A. nidulans* [31].

Other than the induction pathways, carbon catabolite repression (CCR), a mechanism enabling microorganisms to prefer easily metabolizable carbon sources over polymeric or recalcitrant substrates, seems strictly conserved in filamentous fungi [27]. A central mediator of CCR is the zinc-finger TF CreA/Cre1 [33–36], which acts in a double-lock mechanism on both the target genes as well as the regulatory TFs [29]. In *T. reesei* RUT-C30, a truncated version of the *cre1* gene is present [37], leading to a cellulase de-repressed phenotype [35].

The production of cellulases in filamentous fungi is furthermore dependent on the presence of specific inducer molecules. In case of cellulose, the relevant signaling molecules are short cellodextrins such as cellobiose, which are released from cellulose by the action of cellulases, or metabolic derivatives, such as sophorose [38–40].

According to the aforementioned points it is clear, therefore, that multiple factors will affect the production of cellulases in microorganisms: (1) the composition of the substrate, (2) the accessibility of the cellulose to enzymatic attack, (3) the overall enzymatic complement produced by the organism, (4) the nature and amount of inducer molecules being released, and (5) the wiring of the regulatory networks integrating the perceived signals in the respective production organism employed. MCCs as more pure substrates might appear to be less complex in their applicability than plant biomass, but their effectiveness is subject to the same combination of physical, chemical and biological factors. A huge variety of sources, production methods, as well as batch-to-batch variations [16–18, 20] makes it highly demanding for the user to choose the best substrate and warrant studies to determine the most relevant factors. Despite a plethora of studies on the characteristics of cellulose and their effects on enzyme hydrolysis, the effects of central factors, such as crystallinity and fine structure (surface area; porosity), are still unclear and partly disputed [41–51]. However, with some notable exceptions (e.g. [48]) most of these studies used isolated enzyme systems, which is helpful to focus, but is also simplifying, since it ignores the biology of the production organism. To extend our view, we therefore analyzed both the physiochemical and molecular biological aspects of cellulase production in two filamentous fungi when grown on different cellulosic substrates. We chose three representative celluloses: a hardwood- and a softwood-derived MCC as well as a hardwood-derived powdered cellulose, and tested their effectiveness as cellulase-inducing substrates on the hypercellulolytic *T. reesei* strain RUT-C30 and the laboratory model strain *N. crassa*. The physiochemical analyses of the substrates were done at several structural levels (acc. to [45]): fiber (surface area and morphology), fibril (composition, particle size), and microfibril (crystallinity). To assess the fungal performance, cellulase productivity as well as the molecular response were recorded.

## Results

### Substrate characteristics: surface area and morphology

**Fig. 1:**
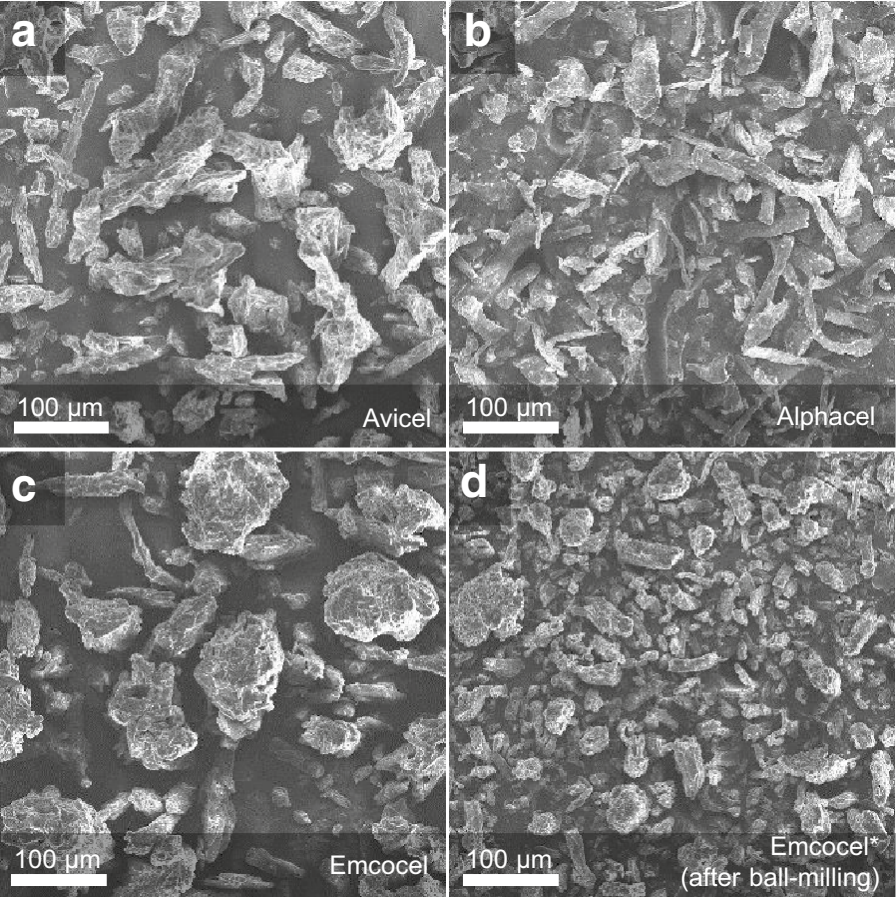
Scanning electron micrographs of cellulose substrates obtained at 8.0 kV accelerating voltage and ×100 magnification (Jeol JSM-IT100). Images show a representative picture for each substrate out of several technical replicates

For this study, three different cellulose products were chosen as cellulase-inducing growth substrates: a hardwood-derived MCC (Emcocel HD90), a softwood-derived MCC (Avicel PH-101) and a hardwood-derived purified cellulose (Alphacel) (see Methods; Table 2). The celluloses were initially observed by scanning electron microscopy (SEM) to visualize macromolecular substrate characteristics. In line with the manufacturer’s specifications, Emcocel contained the largest particles in comparison to the MCC gold standard Avicel as well as the purified cellulose Alphacel (Fig. 1a–c). While Alphacel had the most fibrous appearance (Fig. 1b), Emcocel consisted mostly of particle agglomerates that could be broken up by additional ball-milling (Fig. 1c, d).

N_2_-BET measurements showed an inverse correlation between the specific surface area of the celluloses and the average particle size. The specific surface area of Emcocel (0.80 m^2^/g) was only about 2/3 the area of Avicel (1.28 m^2^/g) and less than half the area of Alphacel (1.64 m^2^/g). After additional ball-milling, however, the surface area of Emcocel doubled and was comparable to the other substrates (1.58 m^2^/g).

### Determination of hemicellulose content of the celluloses

To determine the purity of the different MCCs regarding hemicellulose contaminations, we performed a compositional analysis after total acid hydrolysis. Bacterial cellulose was used as a hemicellulose contamination-free standard for comparison. For Avicel, Alphacel and the bacterial cellulose, a 1 h swelling time in 72% H_2_SO_4_ was sufficient to achieve an almost complete hydrolysis. For Emcocel however, an undissolved residual mass remained after the hydrolysis, indicating that the process had been incomplete (data not shown). For that reason, the hydrolysis of Emcocel was repeated using longer swelling times (4 h) to give the sulfuric acid more time to completely react with the MCC.

As expected, the bacterial cellulose showed no trace of other monosaccharides other than glucose (Table 1). Alphacel on the other hand, was the most unpure cellulose with a particularly high content of xylan. The high xylan:mannan ratio is indicative of its source material being hardwood pulp (acc. to [16]). Avicel as a softwood-derived MCC presented a much more moderate xylan:mannan ratio, but still contained considerable amounts of both hemicelluloses. Emcocel proved to be the least hemicellulose-contaminated cellulose in our analysis with xylan and mannan contents of less than 1% each. This amount was more or less constant at all different swelling times tested (not shown), while the amount of detected glucose increased considerably at 4 h, suggesting that the cellulosic fraction of Emcocel is extremely densely packed and recalcitrant to the hydrolysis. Even after 4 h of swelling, ~ 6–7% of the mass remained unaccounted for. We did not detect elevated amounts of lignin or extractables in Emcocel however (data not shown), indicating that the residual mass is mainly undissolved cellulose.

**Table 1:** Results of sugar analysis of the celluloses after sulfuric acid hydrolysis (in %)

	Avicel^a^	Emcocel^b^	Alphacel^a^	bacterial cellulose^a^
d-glucan	93 ± 5.5	92 ± 0.4	84 ± 5.8	97 ± 4.2
d-xylan	3.5 ± 0.4	0.8 ± 0.08	14.7 ± 1.1	ND
d-mannan	1.8 ± 0.1	0.3 ± 0.01	1.3 ± 0.3	ND

*ND* none determined

^a^1 h swelling

^b^4 h swelling

### Cellulose crystallinity

Crystallinity has been widely used to describe celluloses and woods, since it is a good measure of the inherent degree of structural order and thus may have a major influence on the recalcitrance of the substrate to biochemical attack (e.g. [47, 52–54]). Due to the differences observed in bioavailability of the celluloses, we decided to measure also the crystallinity of all three substrates. To this end, samples of the celluloses were analyzed by solid-state ^13^C nuclear magnetic resonance (NMR) and the crystallinity index (CrI) calculated by the NMR C4 peak separation method [47, 52, 55].

**Fig. 2:**
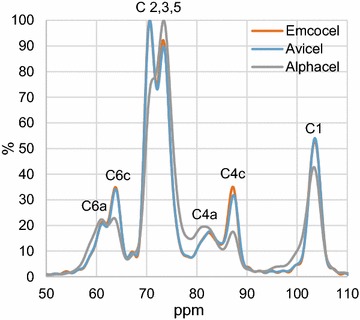
Solid state ^13^C NMR spectra of cellulose samples. Depicted are normalized spectra between 50 and 110 ppm showing the assignment of peaks to the carbons in a glucopyranose repeat unit. Shown is a single but representative spectrum for each cellulose

The NMR spectra displayed noticeable differences in height of the C4 peaks between the samples (Fig. 2). Alphacel showed the lowest crystallinity with 33.1% compared to Avicel with 54.4% and Emcocel with 56.7%. The calculated CrIs therefore allowed for a clear discrimination of the MCCs and the powdered cellulose product Alphacel. Additionally, the results demonstrate that Emcocel was the most crystalline cellulose product in our experiments.

### In vitro digestibility of the cellulose substrates

**Fig. 3:**
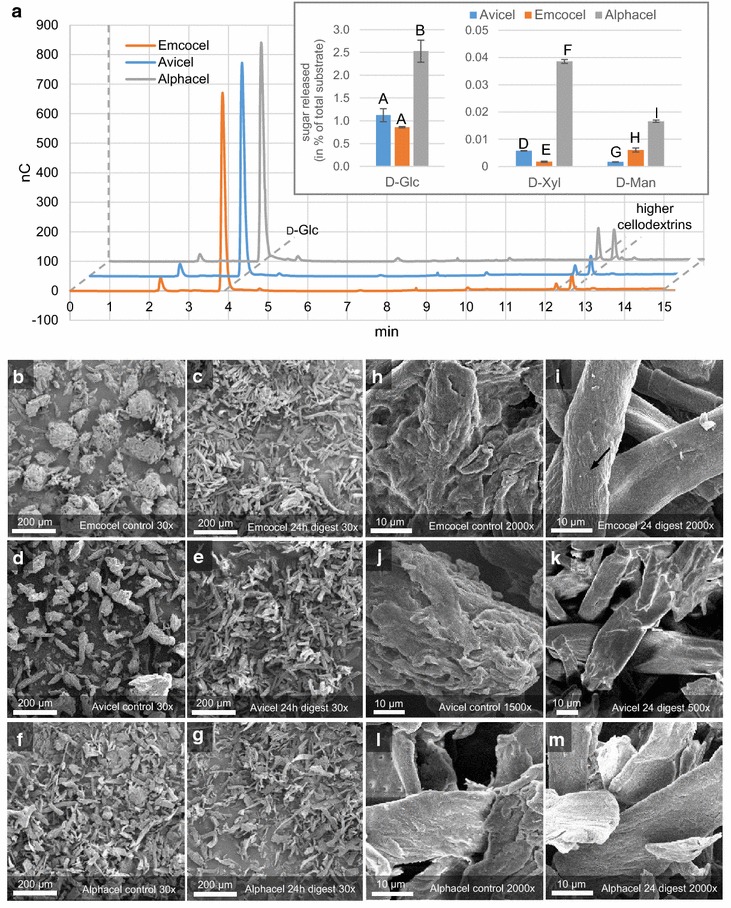
Enzymatic digestion of the cellulose substrates in vitro. All celluloses were digested by a *N. crassa*-derived cellulase cocktail (filtered culture supernatant after 5 days growth on Avicel) for a total of 24 h. **a** Shown are representative HPAEC-PAD chromatograms of the reaction supernatants after 8 h as well as the quantification results for monosaccharides at an initial time point (1 h; inset in a ). The peaks of d-glucose (d-Glc) and higher cellodextrins (not quantified) are indicated. Note: in this run (CarboPac^®^ PA200 column), the other monosaccharides will also migrate at the same speed as d-Glc, but the amounts are substantially lower. The quantifications represent means of triplicate reactions. The error bars represent standard deviations. Letters indicate data groups that are significantly different (one-way ANOVA, *p*-values < 0.05 were considered significant). **b**–**m** Representative scanning electron micrographs out of technical triplicates for each cellulose substrate obtained at 8.0 kV accelerating voltage and × 30–× 2000 magnification (Jeol JSM-IT100)

Since the fungal cellulase induction will be highly dependent on the enzymatic digestibility for the liberation of inducer molecules, we next wanted to test this in an in vitro assay. To this end, *N. crassa* cellulases were incubated with the cellulose substrates. The liberated sugars were analyzed by HPAEC-PAD, and the residual cellulose harvested for SEM analysis (Fig. 3).

The chromatograms indicated that mainly glucose and higher cellodextrins accumulated in the assay supernatants after extensive digestion (8 h; Fig. 3a). The quantified amounts of glucose, xylose and mannose at more initial time points (1 h; Fig. 3a; inset) showed that Alphacel was the most readily digested substrate. Similarly, Emcocel was the least digested substrate again, corroborating that it is most recalcitrant of all three substrates to enzymatic or chemical attack.

When observed by SEM, all three celluloses showed a more particulate appearance after 24 h of enzymatic digestion. This was most prominent for the MCCs, which completely disintegrated into individual fibers (Fig. 3b–g). At higher magnifications, it became evident that the surfaces of all digested substrates appeared smoother than the controls (Fig. 3h–m). This might indicate that the rough top layers were formed by more amorphous cellulose and/or hemicelluloses. It furthermore suggests that the flat layers exposed after the digest might represent more recalcitrant cellulose-rich areas. Particularly the parallel fibrillar structures visible on the surface of some fibers of Emcocel after the digest (Fig. 3i; arrow) seem to represent relatively pure, ordered cellulose fibrils, leaving little contact surface for enzymes to attack.

### Determination of the potential to induce lignocellulolytic gene expression

With a molecular approach, we tested the ability of the cellulose substrates to induce the major cellulolytic and hemicellulolytic pathways on the level of gene expression. Moreover, this analysis was supposed to provide indirect insight into the early bioavailability of inducer molecules, and therefore to what extent fungi will be able to actually perceive the measured differences in the substrate composition. Since the molecular response pathways to cellulose and hemicellulose are more separated in *N. crassa* than in *T. reesei* (see background and reviewed in [27]), we only used *N. crassa* for these assays. Based on a survey of published transcriptomics analyses [31, 38, 56–59], we chose three genes that served as proxies for the induction of the cellulolytic, xylanolytic and mannanolytic pathways in *N. crassa*, since they had been shown to be robustly induced by their respective substrates and serve a specific function in those pathways: the cellobionic acid transporter gene *cbt*-*1*/*clp*-*1* (NCU05853), the d-xylulose kinase encoding gene NCU11353, and the putative acetylmannan esterase encoding gene *ce16*-*1* (NCU09416).

**Fig. 4:**
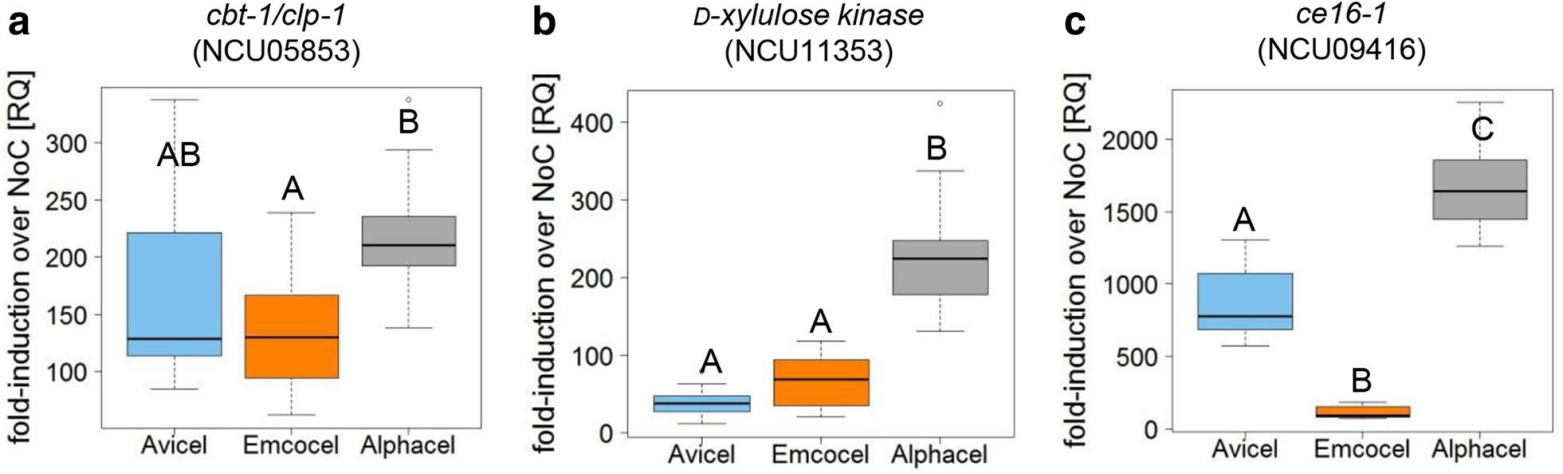
Gene expression induction of selected genes used as proxies for the fungal cellulolytic and hemicellulolytic response. Sucrose pre-grown *N. crassa* cultures were exposed to the celluloses or no carbon source for 4 h before RNA was harvested. Gene induction was measured by RT-qPCR. Shown is the mean fold-induction over the no carbon (No C) starvation condition derived from biological and technical triplicates. Error bars denote standard deviation. Letters indicate data groups that are significantly different (one-way ANOVA, *p*-values < 0.05 were considered significant)

The initial cellulolytic response (as measured by *cbt*-*1*/*clp*-*1*) to Avicel, Emcocel and Alphacel was similar (Fig. 4a). Emcocel, however, showed a tendency to respond weaker. Even though it was found to have the lowest cellulose content of all tested substrates, the response to Alphacel tended to be strongest, indicating a better bioavailability of the cellulose microfibrils.

The xylanolytic response (as measured by the *d*-*xylulose kinase* gene expression) was clearly strongest in the case of Alphacel, which also had the highest content of xylan, and was found to be less intense in the MCC substrates (Fig. 4b). Interestingly however, *N. crassa*’s response to the xylan impurities in Avicel and Emcocel was not necessarily proportional to the overall content. Emcocel induced the xylanolytic pathway more strongly, even though the xylan content was lower than in Avicel, again indicating that the bioavailability is not directly proportional to the overall content.

In case of the mannanolytic pathway, the responses to the three substrates were very distinct (Fig. 4c). Avicel induced the acetylmannan esterase gene *ce16*-*1* > 7-fold stronger than Emcocel, which roughly reflects the difference in mannan content and might be expectable, since Avicel is derived from softwoods and Emcocel from hardwoods, in which xylans are the dominating hemicellulose. The fact that the strongest response was again detected on Alphacel, although the overall mannan content was found to be lower than in Avicel might have two reasons: (1) the bioavailability of the plant cell wall sugars in the non-microcrystalline substrate is generally higher, and (2) there is some evidence that the cellulolytic and mannanolytic pathways cross-react in *N. crassa* (as well as in *Aspergillus oryzae*) [32, 60]. The strong cellulolytic response to Alphacel might therefore also co-induce the mannanolytic pathway more strongly.

### Cellulase production in *N. crassa* and *T. reesei* RUT-C30

**Fig. 5:**
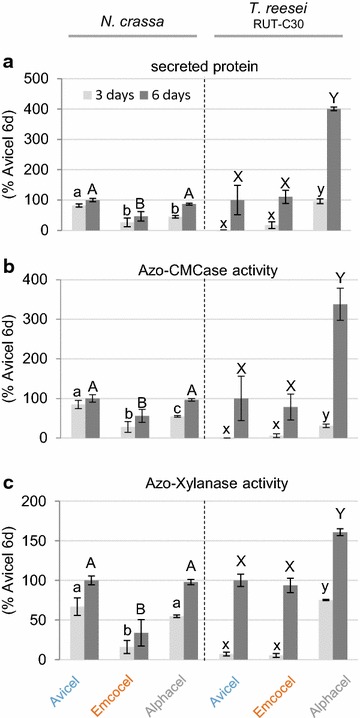
Cellulase and hemicellulase production by *N. crassa* and *T. reesei* RUT-C30 on the cellulose substrates. Performance was measured by analysis of culture supernatant aliquots taken after 3 and 6 days, respectively. Secreted protein was measured by Bradford assay, endo-glucanase activity by Azo-CMC assay and endo-xylanase activity by Azo-Xylanase assay as described in Methods. Values are the mean of biological triplicates. Error bars show standard deviation. Letters indicate data groups that are significantly different (separately for both fungi and sample days; one-way ANOVA, *p*-values < 0.05 were considered significant)

The effectiveness of the three celluloses as substrates for cellulase production in filamentous fungi was tested in 100 ml shake flask cultures. We used both the industrially optimized hypercellulolytic *T. reesei* strain RUT-C30, as well as the genetic model system *N. crassa*. The performance was evaluated after 3 and 6 days of growth by three main analyses of the culture supernatants: total secreted protein concentration, endo-glucanase activity and endo-xylanase activity (Fig. 5). Total and not specific activities (normalized to fungal biomass) are presented, since we aimed to study overall yields on each carbon source. These are therefore representative for the combined effects of bioavailability differences on induction, degradation, metabolism, secretion and growth.

Overall, the data showed that both the softwood-MCC (Avicel) as well as the powdered cellulose (Alphacel) outperformed the hardwood-MCC (Emcocel HD-90) in *N. crassa*, where total secreted protein as well as endo-glucanase and endo-xylanase activities were consistently lowest for Emcocel. While secreted protein, endo-glucanase and endo-xylanase activities were comparable on Avicel and Emcocel (Fig. 5), Alphacel was found to induce cellulases and hemicellulases more strongly in *T. reesei* (Fig. 5b, c). Moreover, protein secretion by *T. reesei* RUT-C30 had a longer lag phase (Fig. 5a; compare day 3 vs. day 6 data).

Since it seemed likely that the poor performance of Emcocel was at least partly due to the much smaller surface area of the substrate (see above), we also tested the performance of the ball-milled Emcocel (Emcocel*; *N. crassa* only) with an about doubled surface area compared to the original (Additional file 1: Fig. S1). Surprisingly however, protein production was almost indistinguishable from the unmilled Emcocel, indicating that the specific surface area was not limiting for the performance in the fermentations.

### WT versus industrially optimized strains

**Fig. 6:**
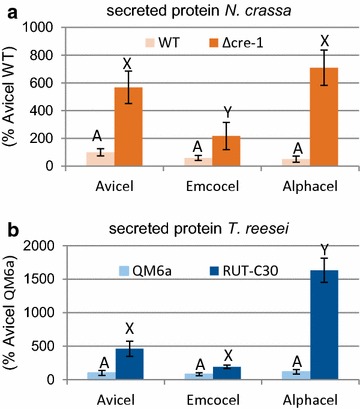
Total protein secreted by *N. crassa* (WT and Δ*cre*-*1*) and *T. reesei* (QM6a and RUT-C30) on the cellulose substrates. Performance was measured by analysis of culture supernatant aliquots taken after 2 and 3 days, respectively. Protein concentration was measured by Bradford assay as described in Methods. Values are the mean of biological triplicates. Error bars show standard deviation. Letters indicate data groups that are significantly different (separately for each strain; one-way ANOVA, *p*-values < 0.05 were considered significant)

Combining the physiochemical properties of the celluloses with the fungal performance data, it appears like hypersecreting systems such as the industrially utilized *T. reesei* RUT-C30 strain are less sensitive to small increases at high crystallinities than non-engineered organisms such as *N. crassa* and are able to take full advantage of the much less crystalline PCs such as Alphacel. We hypothesized that differences in the genetic configuration in these fungi may play a decisive role to this end. Since one of the major modifications from WT to industrial strain is the reduction or removal of CCR, we tested the effect of this by comparing protein production on the three celluloses of the de-repressed *T. reesei* RUT-C30 strain to its WT strain QM6a as well as by comparing the *N. crassa* WT to a deletion strain of *cre*-*1*, encoding the major TF mediating CCR (Fig. 6). To limit the effect of differences in germination speed between the genotypes as much as possible [22, 61], we modified our experimental setup for these assays and shifted identical amounts of fungal biomass to cellulose cultures after pre-growth on sucrose/glucose (see Material and Methods). Since the starting material was mycelia in this experiment, we shortened the incubation time to two and three days for *N. crassa* and *T. reesei,* respectively.

**Fig. 7:**
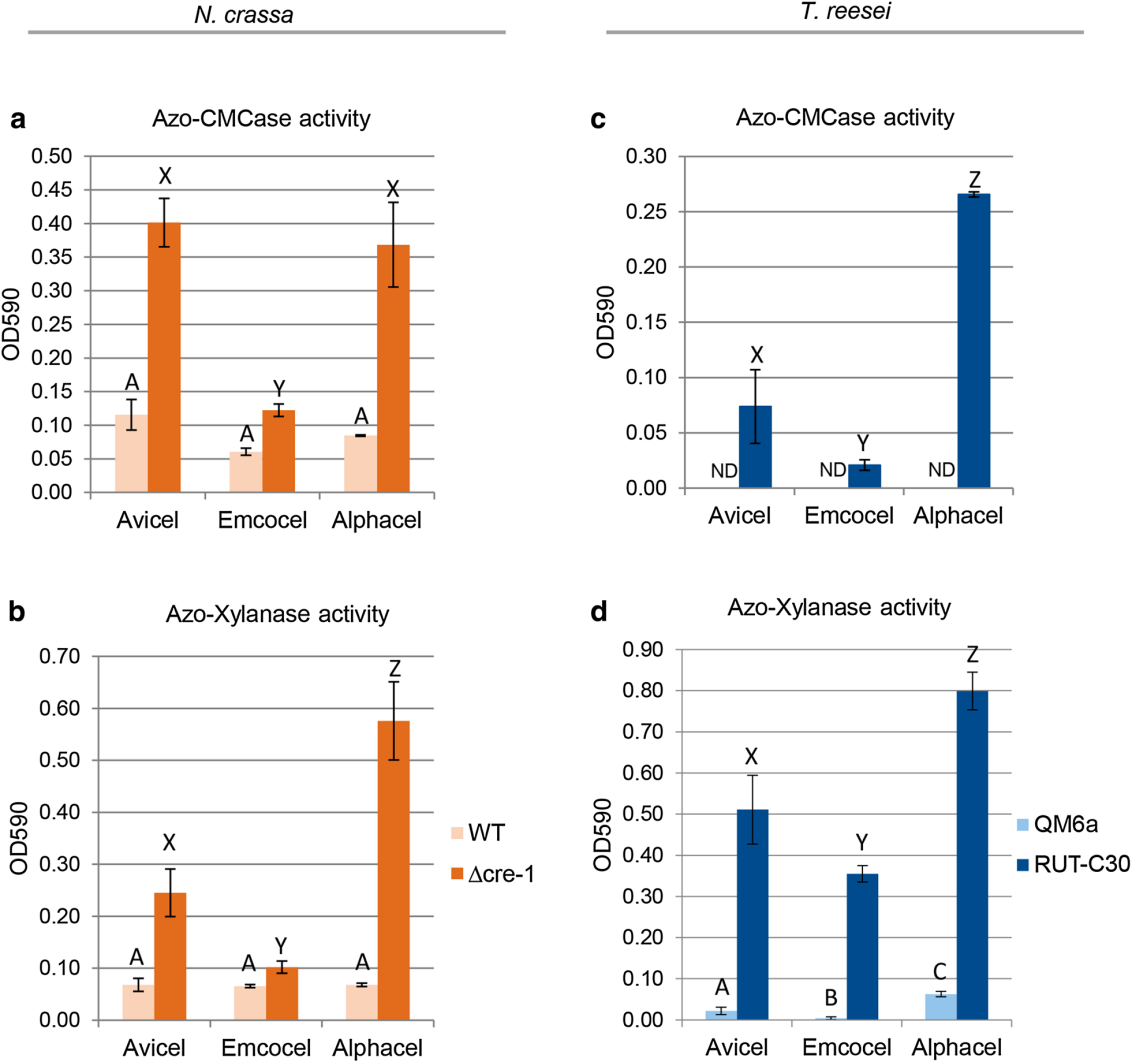
Cellulase and hemicellulase production by *N. crassa* (WT and Δ*cre*-*1*) and *T. reesei* (QM6a and RUT-C30) on the cellulose substrates. Performance was measured by analysis of culture supernatant aliquots taken after 2 and 3 days, respectively. Endo-glucanase activity was measured by Azo-CMC assay and endo-xylanase activity by Azo-Xylanase assay as described in Methods. Values are the mean of biological triplicates. Error bars show standard deviation. Letters indicate data groups that are significantly different (separately for each strain; one-way ANOVA, *p*-values < 0.05 were considered significant). ND indicates that no values could be determined

Two observations could be made: First, the carbon catabolite de-repressed strains (*T. reesei* RUT-C30 with a truncated *cre1* gene [37] and *N. crassa* with a *cre*-*1* deletion) displayed strongly elevated protein levels in comparison to the respective WT strains (Fig. 6) which is in line to what was previously shown in both fungi [36, 62, 63], indicating that CCR has a repressing effect even on highly recalcitrant substrates with low glucose fluxes such as Emcocel (even though the effect there was smallest). And second, also the *T. reesei* WT strain (QM6a) was not able to take full advantage of the low-crystallinity substrates such as Alphacel (Fig. 6b, light blue), just as we had found for *N. crassa* (*cf*. Figure 5). However, while protein production of *T. reesei* RUT-C30 was roughly inversely proportional to the substrate crystallinities again (Fig. 6b, dark blue), the deletion of *cre*-*1* in *N. crassa* was not sufficient to phenocopy this, and protein production on the PC Alphacel was only marginally stronger than on the MCC Avicel (Fig. 6a, darkorange), mainly due to stronger xylanase expression (Fig. 7b) while cellulase expression was similar on the two aforementioned substrates. These observations indicate that other genetic determinants are present in addition to the absence of a fully functional CRE-1 that allows *T. reesei* RUT-C30 to utilize the PC Alphacel better than the other substrates.

## Discussion

In our study, we compared three commercial cellulose powders that might be used as substrates for fungal fermentations regarding their effectiveness as inducers for the production of cellulases and hemicellulases. We chose representative substrates that differed either in their source material (i.e. softwood vs. hardwood) or their production process (i.e. hydrolytically degraded MCC vs. mechanically processed powdered cellulose). Analytical comparisons have shown that powdered celluloses have an overall higher hemicellulose content, lower crystallinity and a broader molecular weight distribution than MCCs [16]. The nature of the source material, on the other hand, can influence properties such as the hemicellulose composition [10, 12, 64]. Depending on the application, one or more of these properties become more relevant than others (e.g. [15, 16]). Consequently, all of these properties might have a strong influence on the performance of the cellulose products in cellulase fermentations. While these points are not well understood and were to be tested here, batch-wise variations were not considered since all chosen substrates were used from a single batch.

The hemicellulosic content of the cellulose products was found to be relevant in the context of the cellulase fermentations. The fungi are able to perceive their presence early on, as demonstrated by their effect on gene expression in *N. crassa* after only 4 h of induction. In agreement and extension of what was proposed by Baehr et al. [16], even the relatively “pure” MCCs can thus be considered to represent heterogeneous systems in which the non-cellulosic parts affect both structure (and thus enzymatic kinetics/accessibility) as well as the fungal response.

The powdered cellulose (Alphacel) was found to be the most readily digested substrate. In agreement with earlier studies, the analytical results confirmed that the crystallinity of the powdered cellulose was substantially lower than that of the MCCs, and that it was furthermore much less pure, with a hemicellulose content of ~ 15% [16]. Also among the MCCs, the softwood-derived product (Avicel) outperformed the hardwood-derived one (Emcocel) when *N. crassa* was used and was found to have a lower crystallinity as well as a higher hemicellulose content. These two factors therefore stand out and appear to be dominant for the effectiveness in our study. Crystallinity and hemicellulose-content might also be linked, since the non-removed hemicelluloses are likely tightly associated with the cellulose by intercalation between the cellulose strands [65], thereby lowering the overall crystallinity.

Intriguingly, enzyme production was inversely proportional to the differences in substrate crystallinity for *T. reesei* RUT-C30, but not for *N. crassa*, where production on Avicel and Alphacel were similar, despite this difference. We hypothesized that in absence of a fully functional CRE-1/Cre1 [24], the higher flux of signaling molecules derived from the more readily digested Alphacel would be directly turned into stronger expression. Indeed, the *T. reesei* WT strain QM6a (with functional CCR) was not able to utilize Alphacel equally well as inducing substrate. However, only xylanase production was found to benefit substantially by deletion of the CCR-mediating TF *cre*-*1* in *N. crassa*, indicating that particularly the XLR-1-dependent hemicellulase production is carbon catabolite repressed in the WT. In the de-repressed mutants, the effect of higher inducing sugar concentrations is then dependent on the regulatory networks present in the fungi [27]. In *T. reesei*, the dominant XYR1 system regulates both cellulase and hemicellulase induction, which might explain why both cellulase and xylanase production are benefiting from the availability of abundant inducing molecules on Alphacel. In *N. crassa* on the other hand, these pathways are much less linked and their regulation is decoupled in a way that CLR-1 and CLR-2 are responsible for the major cellulolytic response while the ortholog of XYR1, XLR-1, is responsible for the xylanolytic response and only modulates the expression of some genes in the cellulose degradation pathway (cf. [31, 58, 66] and reviewed in: [27]). The cross-induction of cellulases by xylan is therefore comparably weak. It is thus feasible that in *N. crassa* the high amount of available xylan in Alphacel will only hyperinduce the XLR-1-dependent hemicellulases, but the inducing effect of the low-crystallinity cellulose is compensated by the lower overall cellulose abundance in Alphacel.

It was also interesting to note that the slightly higher crystallinity of Emcocel compared to Avicel does not seem to allow continuous maximal production capacity despite the similarly strong gene induction measured at 4 h. This seems to indicate that even small differences in measured crystallinity in celluloses manifest themselves in substantial performance differences over time due to a positive feedback loop between substrate bioavailability, metabolism and fungal growth—a fact that has to be considered when choosing a substrate for enzyme fermentations.

Besides the cellular systems, the activity of the enzymes on the substrate for the release of inducer molecules and products will be a bottleneck step. Factors that have been described to affect enzyme activity include: reversible and irreversible adsorption (to cellulose or lignin, respectively), end product inhibition, synergism and others (e.g. [46, 67, 68]). Many studies have looked at the interaction of cellulose structure and enzymatic hydrolysis, and particularly structural characteristics such as crystallinity and surface area have been shown to be determining factors (e.g. [41–46, 48, 49, 51]). It has been pointed out, however, that the effects of crystallinity and surface area are difficult to be evaluated individually, since they are often highly interrelated (at least when celluloses are mechanically (pre-) treated) [45]. In our experiments, surface area did not appear to be limiting, since the performance of Emcocel could not be improved by further ball-milling. This observation is in line with other studies that did not detect any impact of particle size on the rate and extent of sugar release or enzyme binding [42, 45, 69]. However, depending on the fermentation condition and molecular crowding, this might change [68].

Overall, crystallinity seemed to be a major determinant of the effectivity of the celluloses in our study. Digestibility was found to be lower for more crystalline substrates in almost all assays (particularly visible for enzyme production in *T. reesei*). Many previous studies have found that crystallinity critically impacts the enzymatic cellulose hydrolysis (e.g. [41, 43, 44, 49, 51]). We are aware of only one study that was not able to support this finding [50]. Nevertheless, Park et al. cautioned to correlate small differences in CrI, as found between Avicel and Emcocel, with changes in cellulose digestibility, since many other factors might play a role as well, such as for example the distribution of crystalline, para-crystalline and “amorphous” regions in the particles and in relation to the surface area [47]. Still, the resistance of Emcocel towards acid hydrolysis, which was considerably stronger than found for Avicel, confirmed that this product is substantially more recalcitrant and suggests that small differences in CrI might have a strong effect at high crystallinities. The CrI of MCCs might therefore not necessarily correlate linearly with digestibility as previously shown [42] and other variables need to be considered. More studies are needed to determine this in more detail.

## Conclusions

We found that both the inherent cellulose crystallinity and the hemicellulose content are major factors determining the suitability of celluloses as substrates for the expression of cellulases in filamentous fungi as production hosts. Crystallinity restricts the enzymatic digestibility and thus also the release of inducer molecules. Moreover, even in MCCs, that are considered relatively “pure”, the residual hemicellulose content is efficiently perceived by the fungi and—depending on the molecular signaling pathways—translated into a corresponding cellulolytic and hemicellulolytic response, which is not necessarily analogous to the overall composition, indicating differences in bioavailability which cannot be resolved analytically.

The cellulolytic response profiles of *N. crassa* and *T. reesei* were similar, but showed also important differences. For example, only *T. reesei* RUT-C30 was able to take full advantage of the most amorphous cellulose substrate. This effect was not solely due to carbon catabolite de-repression, but is dependent on the species-specific regulatory network. The application of *N. crassa* as an indicator of the activated response pathways therefore seems promising, due to a rapid response time, and since the cellulolytic and hemicellulolytic pathways show a much more limited cross-talk than in *T. reesei* and can thus be individually evaluated.

In conclusion, the presented results provide a set of criteria that can theoretically be applied to broader ranges of substrates and thereby aid in the rational decision for cellulose substrates to be used in enzyme fermentations. Understanding the factors for the performance of cellulose substrates will help to optimize the manufacturing process of lignocellulolytic enzymes from fungi for a number of biotechnological applications, such as for food, feed, textile and biorefinery.

## Methods

### Substrates

Each used substrate is from a single batch preparation. The major physical characteristics of these substrates as indicated by the manufacturers are summarized in Table 2.

**Table 2:** Major physical characteristics of the used substrates (manufacturer’s information)

	Avicel™ PH-101	EMCOCEL^®^ HD90	Alphacel
Brand	Fluka	JRS Pharma GmbH & Co. KG	ICN Biomedicals, Inc.
Type	Softwood MCC	Hardwood MCC	Hardwood PC
Average particle size (µm)	~ 50	124*	35**
Bulk density (g/cm^3^)	0.26–0.31	0.41	0.44–0.50

* By Malvern d50

** Avg. fiber length

### Strains and growth conditions

*N. crassa* wild type (FGSC #2489) and the ∆*cre*-*1* strain (kind gift of N. L. Glass, UC Berkeley, USA) were grown on 2% sucrose Vogel’s minimal medium slants in the dark at 30 °C for 2 days, and transferred to constant light conditions at 25 °C for conidiation. *T. reesei* QM6a and RUT-C30 strains (kind gift of M. Schmoll, AIT, Austria) were propagated on malt extract agar plates in the dark at 28 °C and then switched to 25 °C in constant light for conidiation. The first set of growth experiments (Fig. 5) was performed in flasks containing 100 ml 1% (w/v) cellulose with 1× Vogel’s (at 25 °C and in constant light) or 1× Mandels-Andreotti medium (at 30 °C and in constant light) and at 200 rpm for *N. crassa* and *T. reesei,* respectively [70, 71]. The second set of growth experiments (Figs. 6, 7) was carried out through a two-step cultivation procedure. *N. crassa* and *T. reesei* strains were first grown in 50 ml 2% (w/v) sucrose with 1× Vogel’s for 20 h (at 25 °C and in constant light) or 50 ml 2% (w/v) glucose with 1× Mandels-Andreotti medium for 3 days (at 30 °C and in constant light), respectively. The cultures were collected through vacuum filtration and 0.5 g of *N. crassa* mycelia or 0.42 g of *T. reesei* mycelia were added to 100 ml 1% (w/v) cellulose with 1× Vogel’s or 1× Mandels-Andreotti medium and the cultures were incubated at 25 °C and 200 rpm for 2 days (*N. crassa*) or at 30 °C and 200 rpm for 3 days (*T. reesei*), respectively.

For inoculation, generally a respective volume of conidial suspension was added after optical density measurements in order to achieve a starting concentration of 10^6^ conidia/ml. All assays were done with biological triplicates for each strain per each condition.

### Compositional analyses

Initially, the dried cellulose samples (~ 5 mg) were swollen in 50 µl of 72% H_2_SO_4_ for 1 h (Avicel, Alphacel, bacterial cellulose) or 4 h (ball-milled Emcocel) at RT. After addition of 1.45 ml water, the material was autoclaved for 1 h at 121 °C. Potential residual solids were subsequently removed by centrifugation, and the supernatant analyzed after appropriate dilution using an ICS-3000 liquid chromatography system (Dionex, Thermo Scientific) equipped with a pulsed amperometric detector. The columns used were a CarboPac^®^ PA20 3 × 150 mm (Dionex, Thermo Scientific) and a CarboPac^®^ PA200 3 × 250 mm (Dionex, Thermo Scientific), with column temperature of 30 °C and a flow rate of 0.4 mL/min. Mobile phase for detection of monosaccharides (PA20) was 5 mM NaOH isocratic for 15 min. For detection of cellodextrins (PA200), mobile phases were 0.1 M NaOH (A) and 0.1 M NaOH/1 M NaAc (B). Gradient used was: 2 min 0% B, 10 min 0–10% B and 3 min 0% B. The analyses were done with technical triplicates for each substrate.

### Solid state nuclear magnetic resonance spectroscopy

Solid-state ^13^C NMR spectra were obtained on a Bruker Avance™ III 200 spectrometer (Bruker BioSpin GmbH, Karlsruhe, Germany). Cross-polarization magic angle spinning (CPMAS) was applied with a ^13^C-resonance frequency of 50.32 MHz and a spinning speed of 5 kHz. Contact time was 1 ms and recycle delay was 2 s. Approximately 5000 scans were accumulated and no line broadening was applied. For calibration of the ^13^C chemical shifts, tetramethylsilane was used and set to 0 ppm. Spectral analysis were performed using the spectrometer software. The crystallinity index (CrI) was then calculated by the NMR C4 peak separation method, that assigns peaks at about 87 and 82 ppm in the NMR spectra to the C4 carbons in ordered cellulose structures (“crystalline”; C4c) and non-crystalline domains (“amorphous”; C4a), respectively [47, 52, 55].

We would like to note that the CrI might have been underestimated for Alphacel, since we used a simple drying process instead of solvent-exchange drying, which might have given numbers more representative of the swollen state in the liquid broth. However, the measured differences have found to be less of an issue at high CrIs [41]. The CrIs of the MCCs were thus probably less affected, and that of Avicel was well in line with previously reported values [58].

### Surface area measurements

The specific surface area of the substrates was determined by multi-point BET [72] with an Autosorb-1 analyzer (Quantachrome, Syosset, USA) using nitrogen gas as adsorbate at 77 K. The samples were outgassed before analysis in vacuum under helium flow at 60 °C for 12 h.

### Scanning electron microscopy

All substrates were dried over immobilized on metal stubs using a double sided sticky tape and then sputter coated with gold. The scanning electron micrographs were taken with a JSM-IT100 (JEOL, Freising, Germany) at 8 kV accelerating voltage. The substrates were visualized in triplicates.

### Enzymatic assays

Azo-CMCase and Azo-xylanase activity assays were carried out according to the protocols of the manufacturer (Megazyme, Ireland) (S-ACMC and S-AXBL), slightly modified, since the reaction mixture was reduced to a quarter of the original volume. Assays were done with biological triplicates for each substrate per each strain.

### RNA-extraction and RT-qPCR

Quantification of gene expression was done by quantitative real-time PCR (RT-qPCR) performed on RNA samples that were harvested after a 4 h induction phase on the respective celluloses as described in Benz et al. [49]. The RNA extraction was performed according to the TRIzol Reagent protocol (Fisher Scientific, Schwerte, Germany). RNA was then treated with DNase I (RNase-Free) according to manufacturer’s recommendations (New England Biolabs, Frankfurt am Main, Germany) and subsequently cleaned up with the GeneJET RNA Purification Kit (Fisher Scientific, Schwerte, Germany). A 96-well plate reader (Infinite 200 PRO, Tecan) was used to check RNA purity and concentration. cDNA was obtained following instructions of the High-Capacity cDNA Reverse Transcription Kit (Applied Biosystems; Fisher Scientific, Schwerte, Germany). Finally, RT-qPCR was performed with the sensiFAST SYBR No-ROX Kit (Bioline, Luckenwalde, Germany) on a Mastercycler ep realplex^2^ (Eppendorf, Wesseling-Berzdorf, Germany) and analyzed using the realplex 2.2 software. Actin gene (NCU04173) was used as a reference gene. Primers used are shown in Table 3. Expression analyses were done with biological and technical triplicates for each condition.

**Table 3:** Sequences of primers used for RT-qPCR

Gene	Forward primer	Reverse primer
*actin* (NCU04173)	CATCGACAATGGTTCGGGTATGTG	CCCATACCGATCATGATACCATGATG
*cbt*-*1*/*clp*-*1* (NCU05853)	CGCCCTGACCTACACCTAC	GGCCAAACGACCAAAGAGC
*D*-*xylulose kinase* (NCU11353)	GGCACATCATCGCTTCACTG	CAAGGGAGATGCGCGAGG
*ce16*-*1* (NCU09416)	GGTGCTGCTCCCATCTACTAC	GTACTTGGCGATGGCGAC

### Statistical analyses

Statistical analyses were done by applying analysis of variance (ANOVA) followed by a Tukey test using the statistical computing software R [73].

## Additional file

**Additional file 1.** Cellulase and hemicellulase expression by *N. crassa* and *T. reesei* RUT-C30 on Emcocel with or without additional ball-milling. Performance was measured by analysis of culture supernatant aliquots taken after 3 and 6 days. Secreted protein was measured by Bradford assay, endo-glucanase activity by Azo-CMC assay and endo-xylanase activity by Azo-Xylanase assay as described in Methods. Emcocel* denotes additionally ball-milled substrate. Values are the mean of biological triplicates. Error bars show standard deviation (no statistical differences were detected by one-way ANOVA).

## References

[1] Kuhad RC, Gupta R, Singh A (2011). Microbial cellulases and their industrial applications. Enzyme Res.

[2] Report. Technical enzymes market by type (cellulases, amylases, proteases, lipases, other enzymes), application (bioethanol, paper and pulp, textile and leather, starch processing, other applications), and by region—global forecasts to 2021. Research and markets. 2016.

[3] Habibi Y, Lucia LA, Rojas OJ (2010). Cellulose nanocrystals: chemistry, self-assembly, and applications. Chem Rev.

[4] Himmel ME, Ding S-Y, Johnson DK, Adney WS, Nimlos MR, Brady JW, Foust TD (2007). Biomass recalcitrance: engineering plants and enzymes for biofuels production. Science.

[5] Matthews JF, Skopec CE, Mason PE, Zuccato P, Torget RW, Sugiyama J, Himmel ME, Brady JW (2006). Computer simulation studies of microcrystalline cellulose Iβ. Carbohydr Res.

[6] Pérez S, Samain D, Derek H (2010). Structure and engineering of celluloses. Advances in carbohydrate chemistry and biochemistry.

[7] Nisizawa K (1973). Mode of action of cellulases. J Ferment Technol.

[8] Ding S-Y, Himmel ME (2006). The maize primary cell wall microfibril: a new model derived from direct visualization. J Agric Food Chem.

[9] Somerville C, Bauer S, Brininstool G, Facette M, Hamann T, Milne J, Osborne E, Paredez A, Persson S, Raab T (2004). Toward a systems approach to understanding plant cell walls. Science.

[10] Harada H, Côté WA, Higuchi T (1985). Structure of wood. Biosynthesis and biodegradation of wood components.

[11] Pettersen RC, Rowell R (1984). The chemical composition of wood. The chemistry of solid wood.

[12] Timell TE (1967). Recent progress in the chemistry of wood hemicelluloses. Wood Sci Technol.

[13] Battista OA, Smith PA. Level-off d.p. cellulose products. U.S. Patent No. 2,978,446. 1961.

[14] Hanna M, Biby G, Miladinov V. Production of microcrystalline cellulose by reactive extrusion. U.S. Patent No. 6,228,213. 2001.

[15] Adel AM, Abd El-Wahab ZH, Ibrahim AA, Al-Shemy MT (2011). Characterization of microcrystalline cellulose prepared from lignocellulosic materials. Part II: physicochemical properties. Carbohydr Polym.

[16] Baehr M, Führer C, Puls J (1991). Molecular weight distribution, hemicellulose content and batch conformity of pharmaceutical cellulose powders. Eur J Pharm Biopharm.

[17] Landín M, Martínez-Pacheco R, Gómez-Amoza JL, Souto C, Concheiro A, Rowe RC (1993). Effect of batch variation and source of pulp on the properties of microcrystalline cellulose. Int J Pharm.

[18] Landín M, Martínez-Pacheco R, Gómez-Amoza JL, Souto C, Concheiro A, Rowe RC (1993). Effect of country of origin on the properties of microcrystalline cellulose. Int J Pharm.

[19] Newman RH (1994). Crystalline forms of cellulose in softwoods and hardwoods. J Wood Chem Technol.

[20] Rowe RC, McKillop AG, Bray D (1994). The effect of batch and source variation on the crystallinity of microcrystalline cellulose. Int J Pharm.

[21] Paloheimo M, Haarmann T, Mäkinen S, Vehmaanperä J, Schmoll M, Dattenböck C (2016). Production of industrial enzymes in *Trichoderma reesei*. Gene expression systems in fungi: advancements and applications.

[22] Seiboth B, Ivanova C, Seidl-Seiboth V, Dos Santos Bernardes MA (2011). *Trichoderma reesei*: a fungal enzyme producer for cellulosic biofuels. Biofuel production—recent developments and prospects.

[23] Viikari L, Vehmaanperä J, Koivula A (2012). Lignocellulosic ethanol: from science to industry. Biomass Bioenergy.

[24] Le Crom S, Schackwitz W, Pennacchio L, Magnuson JK, Culley DE, Collett JR, Martin J, Druzhinina IS, Mathis H, Monot F (2009). Tracking the roots of cellulase hyperproduction by the fungus *Trichoderma reesei* using massively parallel DNA sequencing. Proc Natl Acad Sci U S A.

[25] Peterson R, Nevalainen H (2012). *Trichoderma reesei* RUT-C30—thirty years of strain improvement. Microbiology.

[26] Kubicek CP (2013). Systems biological approaches towards understanding cellulase production by *Trichoderma reesei*. J Biotechnol.

[27] Glass NL, Schmoll M, Cate JH, Coradetti S (2013). Plant cell wall deconstruction by ascomycete fungi. Annu Rev Microbiol.

[28] Häkkinen M, Valkonen MJ, Westerholm-Parvinen A, Aro N, Arvas M, Vitikainen M, Penttilä M, Saloheimo M, Pakula TM (2014). Screening of candidate regulators for cellulase and hemicellulase production in *Trichoderma reesei* and identification of a factor essential for cellulase production. Biotechnol Biofuels.

[29] Huberman LB, Liu J, Qin L, Glass NL (2016). Regulation of the lignocellulolytic response in filamentous fungi. Fungal Biol Rev.

[30] Seibert T, Thieme N, Benz JP, Schmoll M, Dattenböck C (2016). The renaissance of *Neurospora crassa*: how a classical model system is used for applied research. Gene expression systems in fungi: advancements and applications.

[31] Coradetti ST, Craig JP, Xiong Y, Shock T, Tian C, Glass NL (2012). Conserved and essential transcription factors for cellulase gene expression in ascomycete fungi. Proc Natl Acad Sci U S A.

[32] Craig JP, Coradetti ST, Starr TL, Glass NL (2015). Direct target network of the *Neurospora crassa* plant cell wall deconstruction regulators CLR-1, CLR-2, and XLR-1. MBio.

[33] Bailey C, Arst HN (1975). Carbon catabolite repression in *Aspergillus nidulans*. Eur J Biochem.

[34] de la Serna I, Ng D, Tyler BM (1999). Carbon regulation of ribosomal genes in *Neurospora crassa* occurs by a mechanism which does not require Cre-1, the homologue of the *Aspergillus* carbon catabolite repressor, CreA. Fungal Genet Biol.

[35] Ilmén M, Thrane C, Penttilä M (1996). The glucose repressor gene *cre1* of *Trichoderma*: isolation and expression of a full-length and a truncated mutant form. Mol Gen Genet MGG.

[36] Sun J, Glass NL (2011). Identification of the CRE-1 cellulolytic regulon in *Neurospora crassa*. PLoS ONE.

[37] Mello-de-Sousa TM (2014). A truncated form of the carbon catabolite repressor 1 increases cellulase production in *Trichoderma reesei*. Biotechnol Biofuels.

[38] Sternberg D, Mandels GR (1979). Induction of cellulolytic enzymes in *Trichoderma reesei* by sophorose. J Bacteriol.

[39] Zhou Q, Xu J, Kou Y, Lv X, Zhang X, Zhao G, Zhang W, Chen G, Liu W (2012). Differential involvement of β-glucosidases from *Hypocrea jecorina* in rapid induction of cellulase genes by cellulose and cellobiose. Eukaryot Cell.

[40] Znameroski EA, Coradetti ST, Roche CM, Tsai JC, Iavarone AT, Cate JH, Glass NL (2012). Induction of lignocellulose-degrading enzymes in *Neurospora crassa* by cellodextrins. Proc Natl Acad Sci U S A.

[41] Fan LT, Lee YH, Beardmore DR (1981). The influence of major structural features of cellulose on rate of enzymatic hydrolysis. Biotechnol Bioeng.

[42] Fan LT, Lee Y-H, Beardmore DH (1980). Mechanism of the enzymatic hydrolysis of cellulose: effects of major structural features of cellulose on enzymatic hydrolysis. Biotechnol Bioeng.

[43] Hall M, Bansal P, Lee JH, Realff MJ, Bommarius AS (2010). Cellulose crystallinity—a key predictor of the enzymatic hydrolysis rate. FEBS J.

[44] Li L, Zhou W, Wu H, Yu Y, Liu F, Zhu D (2014). Relationship between crystallinity index and enzymatic hydrolysis performance of celluloses separated from aquatic and terrestrial plant materials. BioResources.

[45] Mansfield SD, Mooney C, Saddler JN (1999). Substrate and enzyme characteristics that limit cellulose hydrolysis. Biotechnol Prog.

[46] Mooney CA, Mansfield SD, Beatson RP, Saddler JN (1999). The effect of fiber characteristics on hydrolysis and cellulase accessibility to softwood substrates. Enzyme Microb Technol.

[47] Park S, Baker JO, Himmel ME, Parilla PA, Johnson DK (2010). Cellulose crystallinity index: measurement techniques and their impact on interpreting cellulase performance. Biotechnol Biofuels.

[48] Peciulyte A, Anasontzis GE, Karlström K, Larsson PT, Olsson L (2014). Morphology and enzyme production of *Trichoderma reesei* Rut C-30 are affected by the physical and structural characteristics of cellulosic substrates. Fungal Genet Biol.

[49] Peng H, Li H, Luo H, Xu J (2013). A novel combined pretreatment of ball milling and microwave irradiation for enhancing enzymatic hydrolysis of microcrystalline cellulose. Bioresour Technol.

[50] Puri VP (1984). Effect of crystallinity and degree of polymerization of cellulose on enzymatic saccharification. Biotechnol Bioeng.

[51] Rollin JA, Zhu Z, Sathitsuksanoh N, Zhang YHP (2011). Increasing cellulose accessibility is more important than removing lignin: a comparison of cellulose solvent-based lignocellulose fractionation and soaking in aqueous ammonia. Biotechnol Bioeng.

[52] Horii F, Hirai A, Kitamaru R (1984). CP/MAS carbon-13 NMR study of spin relaxation phenomena of cellulose containing crystalline and noncrystalline components. J Carbohydr Chem.

[53] Newman RH (2004). Homogeneity in cellulose crystallinity between samples of *Pinus radiata* wood. Holzforschung.

[54] Sterk H, Sattler W, Janosi A, Paul D, Esterbauer H (1987). Einsatz der Festkörper 13C-NMR-Spektroskopie für die Bestimmung der kristallinität in Cellulosen. Das Papier.

[55] Newman RH, Hemmingson JA (1990). Determination of the degree of cellulose crystallinity in wood by carbon-13 nuclear magnetic resonance spectroscopy. Holzforschung.

[56] Benz JP, Chau BH, Zheng D, Bauer S, Glass NL, Somerville CR (2014). A comparative systems analysis of polysaccharide-elicited responses in *Neurospora crassa* reveals carbon source-specific cellular adaptations. Mol Microbiol.

[57] Ogawa M, Kobayashi T, Koyama Y (2012). ManR, a novel Zn(II)2Cys6 transcriptional activator, controls the beta-mannan utilization system in *Aspergillus oryzae*. Fungal Genet Biol.

[58] Sun J, Tian C, Diamond S, Glass NL (2012). Deciphering transcriptional regulatory mechanisms associated with hemicellulose degradation in *Neurospora crassa*. Eukaryot Cell.

[59] Tian C, Beeson WT, Iavarone AT, Sun J, Marletta MA, Cate JH, Glass NL (2009). Systems analysis of plant cell wall degradation by the model filamentous fungus *Neurospora crassa*. Proc Natl Acad Sci U S A.

[60] Ogawa M, Kobayashi T, Koyama Y (2013). ManR, a transcriptional regulator of the beta-mannan utilization system, controls the cellulose utilization system in *Aspergillus oryzae*. Biosci Biotechnol Biochem.

[61] Dashtban M, Buchkowski R, Qin W (2011). Effect of different carbon sources on cellulase production by Hypocrea jecorina (*Trichoderma reesei*) strains. Int J Biochem Mol Biol.

[62] Portnoy T, Margeot A, Linke R, Atanasova L, Fekete E, Sándor E, Le Crom S (2011). The CRE1 carbon catabolite repressor of the fungus *Trichoderma reesei*: a master regulator of carbon assimilation. BMC Genom.

[63] Tangnu SK, Blanch HW, Wilke CR (1981). Enhanced production of cellulase, hemicellulase, and β-glucosidase by *Trichoderma reesei* (Rut C-30). Biotechnol Bioeng.

[64] Willför S, Sundberg A, Pranovich A, Holmbom B (2005). Polysaccharides in some industrially important hardwood species. Wood Sci Technol.

[65] O’Sullivan AC (1997). Cellulose: the structure slowly unravels. Cellulose.

[66] Mach-Aigner AR, Pucher ME, Steiger MG, Bauer GE, Preis SJ, Mach RL (2008). Transcriptional regulation of xyr1, encoding the main regulator of the xylanolytic and cellulolytic enzyme system in *Hypocrea jecorina*. Appl Environ Microbiol.

[67] Fox JM, Levine SE, Clark DS, Blanch HW (2012). Initial- and processive-cut products reveal cellobiohydrolase rate limitations and the role of companion enzymes. Biochemistry.

[68] Levine SE, Fox JM, Blanch HW, Clark DS (2010). A mechanistic model of the enzymatic hydrolysis of cellulose. Biotechnol Bioeng.

[69] Peters LE, Walker LP, Wilson DB, Irwin DC (1991). The impact of initial particle size on the fragmentation of cellulose by the cellulase of *Thermomonospora fusca*. Bioresour Technol.

[70] Mandels M, Andreotti R (1978). Problems and challenges in the cellulose to cellulase fermentation. Proc Biochem.

[71] Vogel HJ (1956). A convenient growth medium for Neurospora (medium N). Microb Genet Bull.

[72] Brunauer S, Emmett PH, Teller E (1938). Adsorption of gases in multimolecular layers. J Am Chem Soc.

[73] Development Core Team. R: a language and environment for statistical computing. R Foundation for Statistical Computing, Vienna, Austria. 2013.

