# Supplementary figures and images for: Comparing the physiochemical parameters of three celluloses reveals new insights into substrate suitability for fungal enzyme production
Source: Fungal Biol Biotechnol. 2017 Nov 3;4:10. doi: 10.1186/s40694-017-0039-9 (PMC5669031; doi:10.1186/s40694-017-0039-9)

Figure S1

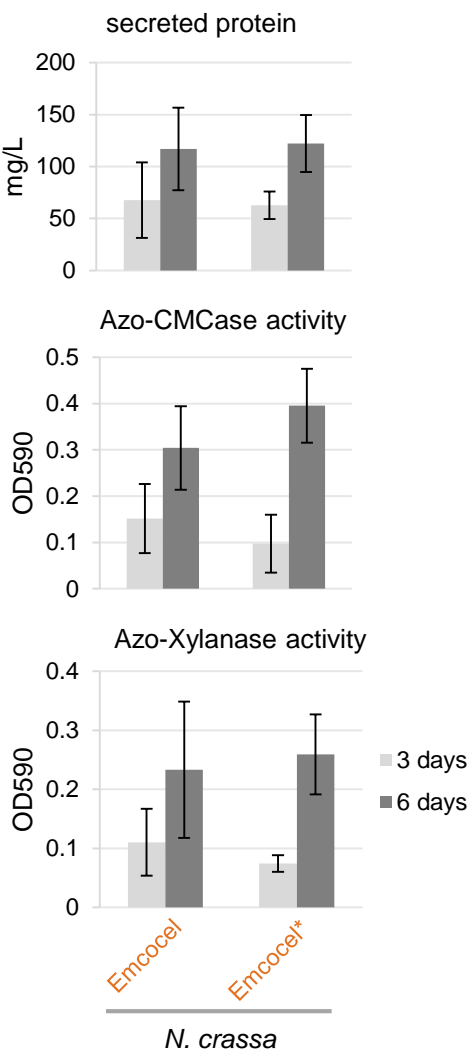

Supplement: Supplementary file 1 — Additional file 1. Cellulase and hemicellulase expression by N. crassa and T. reesei RUT-C30 on Emcocel with or without additional ball-milling. Performance was measured by analysis of culture supernatant aliquots taken after 3 and 6 days. Secreted protein was measured by Bradford assay, endo-glucanase activity by Azo-CMC assay and endo-xylanase activity by Azo-Xylanase assay as described in Methods. Emcocel* denotes additionally ball-milled substrate. Values are the mean of biological triplicates. Error bars show standard deviation (no statistical differences were detected by one-way ANOVA). [file 40694_2017_39_MOESM1_ESM.pdf]
